# Surveillance and phylogenetic analysis of a pathogenic bacterium candidate in nasal discharge from children

**DOI:** 10.1128/spectrum.00566-24

**Published:** 2024-05-24

**Authors:** Kazumasa Fukuda, Kaoru Haro, Kei Yamasaki, Hiroaki Ikegami, Mitsumasa Saito

**Affiliations:** 1 Department of Microbiology, University of Occupational and Environmental Health, Japan, Kitakyushu, Fukuoka; 2 Department of Respiratory Medicine, University of Occupational and Environmental Health, Japan, Kitakyushu, Fukuoka; Yale School of Public Health, New Haven, Connecticut, USA

**Keywords:** uncultured bacterium, 16S rRNA gene, infectious disease

## Abstract

“The infectious organism lurking in human airways (IOLA)” is a candidate pathogenic bacterium detected in bronchoalveolar lavage fluid specimens derived from adult patients with chronic lower respiratory tract infections. Genomic analyses of IOLA have revealed that it possesses the smallest and most AT-rich genome among human-derived bacteria. However, its biological properties remain unclear because no culture method has been established for IOLA. Here, we conducted a large-scale IOLA surveillance study of nasal discharge specimens from children in Japan and investigated the correlation between IOLA detection frequency and patient characteristics. We detected IOLA in 5.4% (103 of 1,920) of pediatric nasal discharge samples. No significant differences were observed in the frequency of detection based on the patient’s background. However, with respect to age, the frequency of detection tended to be significantly high at 2–3 and 6 years old. Phylogenetic analysis revealed five phylotypes in the IOLA 16S rRNA gene sequences, and the sequences detected in adult patients with respiratory infections in a previous study belonged to one of the five phylotypes. The involvement of IOLA in the symptoms is not clear, but IOLA is detected at a relatively high frequency in pediatric nasal discharge. Many subjects with detected IOLA were not always IOLA positive, and IOLA was detected transiently. Our findings suggest that IOLA is horizontally transmitted through groups in nursery and elementary schools, and there are differences in biological characteristics among the IOLA phylotypes.

**IMPORTANCE:**

“The infectious organism lurking in human airways (IOLA)” is a candidate pathogenic bacterium strongly suspected to be infectious to the respiratory tracts of humans and animals. However, a culture method for IOLA has not been established yet, and its properties remain unclear. In this study, IOLA was detected at a relatively high frequency in the nasal discharge of children, and five phylotypes of IOLA were identified. One of these phylotypes was found in the bronchoalveolar lavage fluid from adult patients, suggesting lineage-specific differences in the pathogenicity of IOLA. Moreover, it was suggested that IOLA is horizontally transmitted when children gather in groups such as nursery and elementary schools. These findings strongly indicate that IOLAs have been clinically undetected so far but are spreading among children, with one lineage being involved in respiratory diseases in adults. Examining the presence of IOLA in clinical specimens may help to understand the etiology of respiratory diseases with unknown causes.

## INTRODUCTION

Our understanding of microbial communities in various environments has been improved by advancements in culture-independent analysis methods owing to technical innovations in DNA sequencing technologies (1). As a result, many novel bacterial lineages have been discovered at the phylum level in these environments (2–4). Surveillances of the microbiome in the human body have also been conducted (5–9). Between 2010 and 2020, 197 novel bacteria were reported to be pathogenic to humans (10). However, novel bacteria at taxonomic levels higher than genera are extremely rare. Previously, we discovered a peculiar bacterium that predominates in the bronchoalveolar lavage fluid (BALF) specimens from patients with respiratory tract disorders (11), temporarily named “infectious organism lurking in human airways (IOLA).” IOLA possesses the smallest (303,838 bp) bacterial genome with the lowest GC content (20.7%) among known human-associated bacteria and is comparable to the genomes of insect endosymbionts (12). In addition, genome-based phylogenetic analyses have revealed that IOLA represents a novel lineage at the family level of Alphaproteobacteria, which is genetically distant from existing bacterial lineages (12). In humans, IOLA-like 16S rRNA sequences have been detected only in clinical specimens from patients with respiratory disorders (12, 13) and are predominantly detected in the lungs of patients with signs of a bacterial respiratory infection. However, no known pathogens have been detected, and IOLA can persist in the human respiratory tract for at least 15 months (12). Furthermore, IOLA-like 16S rRNA sequences have also been detected in tracheal samples from a bioengineered lung-transplanted swine (14). These findings suggest that IOLA may be a bacterium associated with human and animal diseases. Investigating and ascertaining the biological properties of IOLA is an urgent issue; however, since no culture method has been established, its pathogenicity and relevance to humans are still unknown. During the microbiota survey of various clinical specimens, IOLA 16S rRNA sequences were also frequently detected in pediatric nasal discharge specimens (4/40) (15). Nasal discharge specimens are aspirated as part of the treatment and can be collected noninvasively, making them suitable for large-scale investigations. Furthermore, it is also necessary to elucidate the dynamics and role of IOLA in nasal discharge specimens from children to comprehend its intrinsic properties. Therefore, we conducted a large-scale IOLA surveillance study of pediatric nasal discharge specimens and investigated the correlation between IOLA detection frequency and patient characteristics.

## RESULTS

### Patient characteristics

A total of 1,920 nasal discharge specimens were collected at pediatric clinics in Kitakyushu City and Fukuoka City over a 2-year period (960 specimens each). There were 1,162 patients overall: 641 in Kitakyushu City and 521 in Fukuoka City. Patient characteristics are shown in Table 1; Table S1. Patient ages ranged from 0 to 12 years with a mean of 1.04 years. The male-to-female ratio was 47.8:52.2. A total of 449 specimens (23.4%) were obtained from patients with a fever of 38°C or higher at the time of admission. A total of 176 specimens (9.2%) were obtained from patients taking antibiotics 1 month prior to the clinic visit. Patients were most frequently diagnosed with acute respiratory tract inflammation (85.4%), followed by respiratory syncytial virus infection (7.1%), acute pneumonia (2.6%), influenza (1.8%), adenovirus infection (0.8%), human metapneumovirus infection (0.7%), allergic rhinitis (0.4%), and streptococcal infections (0.3%). The diagnostic results of fewer than five specimens (including nasal congestion, hand-foot-and-mouth disease, rotavirus gastroenteritis, Kawasaki disease, and exanthema subitum) were classified as others (0.9%).

**TABLE 1:** Clinical characteristics of the patients with nasal discharge symptoms^*a*^

Characteristics	Total	IOLA-specific PCR	
		Positive	Negative
Sample, *n*	1,920	103	1,817
Patient, *n*	1,162	88	1,074
Age (year), median (range)	1 (0–12)	1 (0–6)	1 (0–12)
Age (year), average	1.04	2.40	1.02
Sex (male), *n* (%)	1,002 (52.2)	48 (46.6)	954 (52.5)
Antimicrobial drug use, *n* (%)	176 (9.2)	15 (14.6)	161 (8.9)
Fever, *n* (%)	449 (23.4)	20 (19.4)	429 (23.6)
Diagnosis			
Acute respiratory tract inflammation, *n* (%)	1,639 (85.4)	95 (92.2)	1,544 (85.0)
Acute pneumonia, *n* (%)	50 (2.6)	2 (1.9)	48 (2.6)
Respiratory syncytial virus infection, *n* (%)	136 (7.1)	3 (2.9)	133 (7.3)
Influenza virus infection, *n* (%)	35 (1.8)	3 (2.9)	32 (1.8)
Adenovirus infection, *n* (%)	16 (0.8)	0 (0.0)	16 (0.9)
Human metapneumovirus, *n* (%)	14 (0.7)	0 (0.0)	14 (0.8)
Streptococcal infection, *n* (%)	5 (0.3)	0 (0.0)	5 (0.3)
Allergic rhinitis, *n* (%)	8 (0.4)	0 (0.0)	8 (0.4)
Others, *n* (%)	17 (0.9)	0 (0.0)	17 (0.9)

^*a*^ Patients aged less than 1 year were defined as being 0 years old. Fever was defined as a temperature of 38°C or higher. The diagnostic results for fewer than five specimens were classified as “others.”

### Detection of IOLA-16S rRNA gene

**Fig 1:**
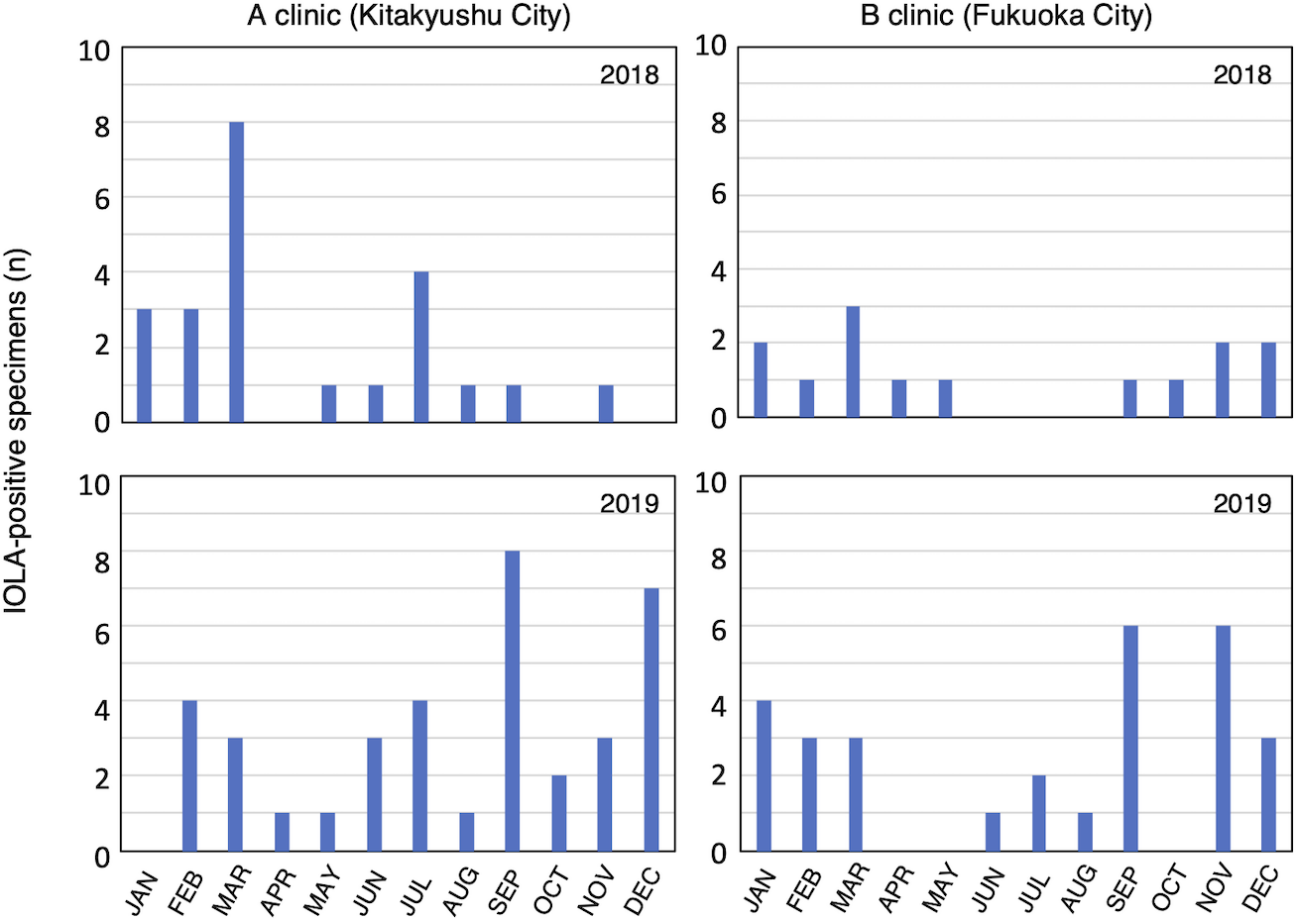
Comparison of the numbers of IOLA-positive specimens detected in each month and each clinic. The numbers of specimen with IOLA-positive PCR result were shown. The upper row represents the results for 2018, while the lower row represents the results for 2019. The left column represents the results for clinic A in Kitakyushu City, and the right column represents the results for clinic B in Fukuoka City. A total of 40 specimens were examined per month at each clinic.

**Fig 2:**
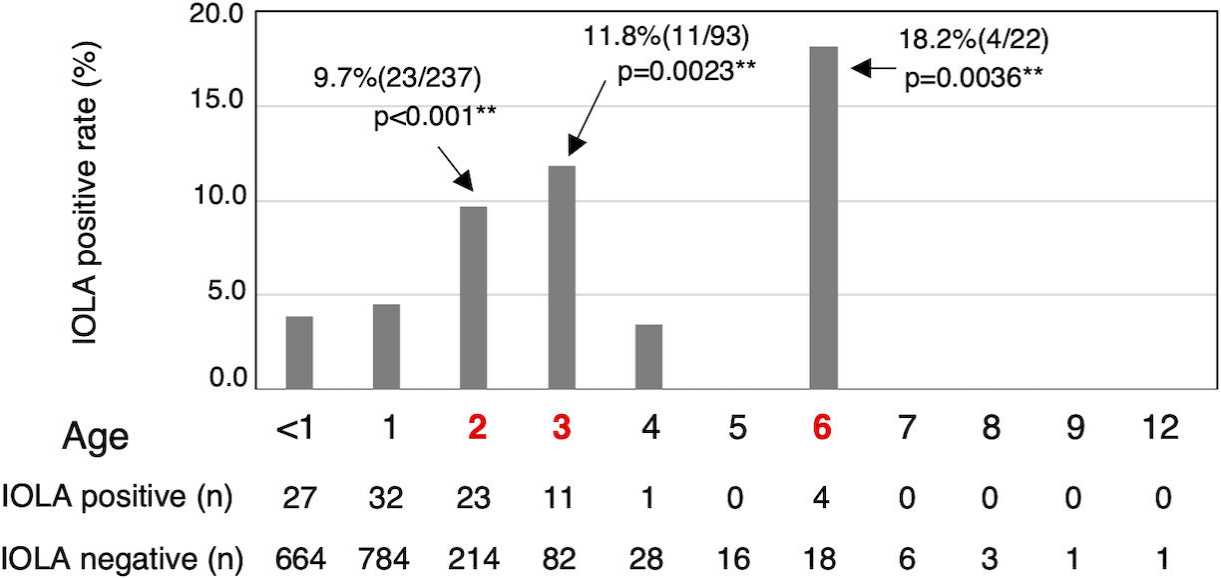
Frequency of IOLA-specific PCR-positive specimens by patient age. The difference between the numbers of IOLA-positive and IOLA-negative patients with respect to patient age was confirmed by Pearson’s χ^2^ test (*P* < 0.01). Gray boxes indicate the IOLA-positive rate for each age group. The numbers indicate the positive rate and residual analysis results (two-sided *P* value), and the numbers in parentheses indicate the number of specimens (IOLA-positive/total specimens). Red numbers indicate ages with significantly higher IOLA-positive rates. The number of IOLA-positive and IOLA-negative specimens at each age is also shown.

The IOLA 16S rRNA gene was detected in 103 specimens with a detection rate of 5.4%. The detection rates at each clinic were 6.3% in Kitakyushu City and 4.5% in Fukuoka City. Figure 1 shows the frequency of IOLA detection according to the month. No seasonal changes in IOLA detection rates were observed. No statistically significant differences were found in IOLA detection rates according to sex (*P* = 0.243), presence or absence of fever (*P* = 0.328), or antibiotic use (*P* = 0.051; Table 2). However, a significant difference (*P* < 0.001) in age between IOLA-positive and IOLA-negative patients was observed (Table 2). Furthermore, the Mann–Whitney U test was performed on the IOLA-positive frequency by age, demonstrating a significant difference. Figure 2 shows the IOLA detection rate based on the age structure of the patients and the results of the residual analysis. The results showed that the frequency of IOLA detection differed significantly by age, with IOLA-positive rates tending to be higher at ages 2–3 and at 6 years old (Fig. 2). There was no significant correlation between the diagnostic results and IOLA detection. However, when the diagnostic results were divided into acute respiratory tract inflammation of unclear cause and other results with clear causes, the IOLA positive rate was significantly higher (*P* = 0.043) in acute respiratory tract inflammation (Table 2).

**TABLE 2:** Comparison of frequency of positive IOLA-specific PCR results on patient characteristics

	IOLA PCR		
	Positive	Negative	*P* value
Age^*a*^	2.40 (±1.35)	1.02 (±1.22)	*P* < 0.001**
Sex			
Male	48	954	*P* = 0.243
Female	55	863	
Fever			
≧38°C	20	429	*P* = 0.328
<38°C	83	1,388	
Antibiotics			
Used	15	161	*P* = 0.051
Not used	88	1,656	
Diagnosis			
Acute respiratory tract inflammation	95	1,544	*P* = 0.043*
Other than acute respiratory tract inflammation	8	273	
Location (city)			
Kitakyushu	60	900	*P* = 0.085
Fukuoka	43	917	

^*a*^ Mann–Whitney U test was performed for the ages, and each average is shown. For all others, Pearson’s χ^2^ test was performed, and the number of IOLA-positive and IOLA-negative specimens is shown. Two-sided *P* values < 0.05 were considered statistically significant. *P* values: *, <0.05; **, <0.01.

### IOLA 16S rRNA gene sequence analysis

**Fig 3:**
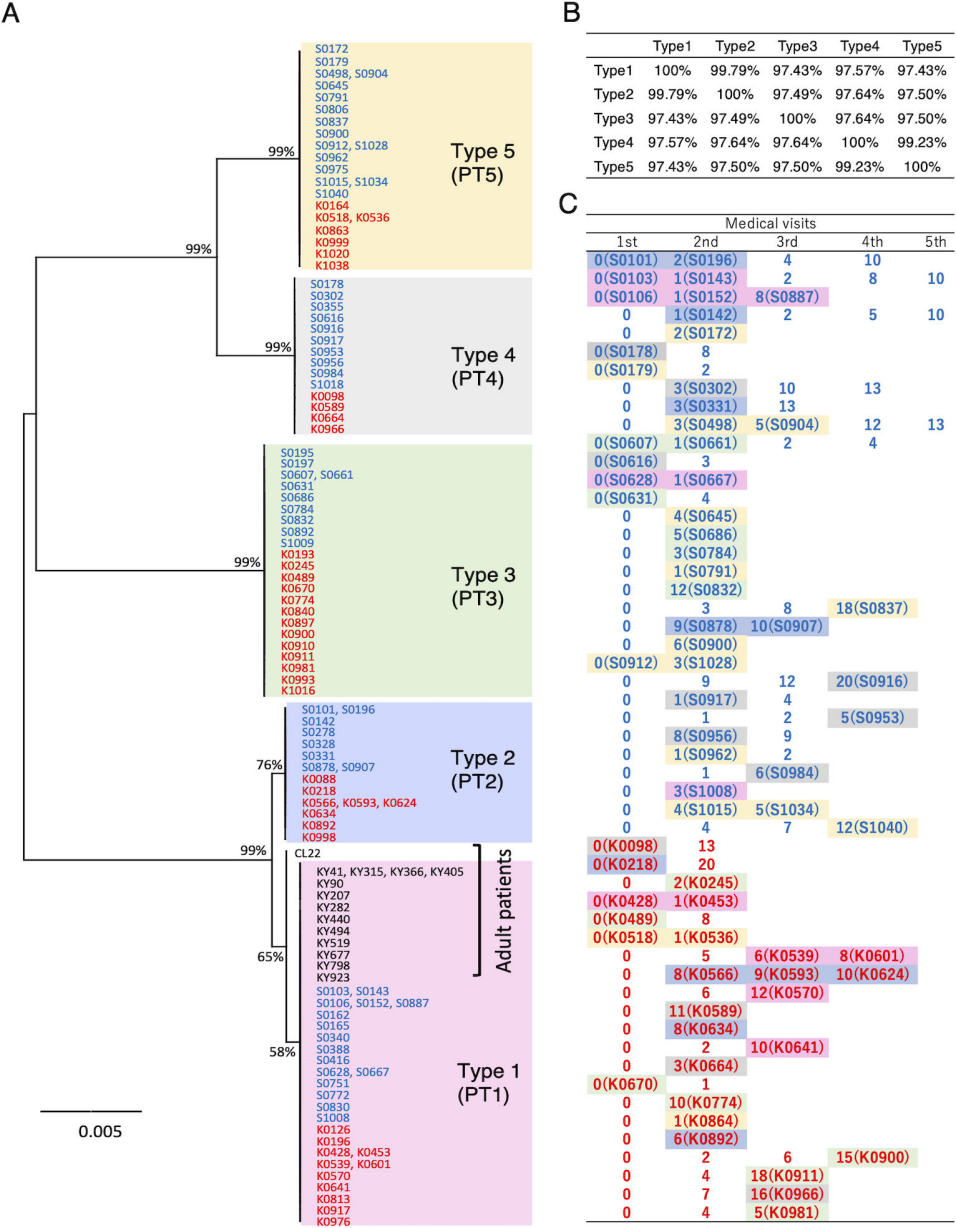
Phylogenetic analysis of IOLA-16S rRNA gene sequences obtained from nasal discharge of children and from BALF specimens of adult patients with respiratory diseases. (**A**) ML phylogenetic tree of IOLA-16S rRNA gene sequences. Sample names of nasal discharge obtained from children Kitakyushu City are shown in blue, and samples obtained in Fukuoka City are shown in red. Black letters indicate specimen names obtained from the BALF of adult respiratory disease patients obtained in previous studies. Bootstrap analysis results are shown beside each branch. (**B**) Sequence similarity profile for each IOLA phylotype. (**C**) Transition of IOLA-specific PCR results in IOLA-positive patients who visited the hospital multiple times. Numbers indicate the number of months that have passed since the first visit was set to 0. Numbers in parentheses indicate sample numbers. Colored boxes indicate IOLA-positive specimens, and color indicates each phylotype.

Amplified products were obtained by PCR in all 103 IOLA-positive samples, and the high-precision nucleotide sequence between the primers IOLA27F and IOLAnR2 (approximately 1,440 bp) was determined. Figure 3A shows a maximum likelihood (ML) tree constructed using the obtained 103 sequences and the 14 IOLA16S sequences derived from adult lung disease patients obtained in a previous study. The IOLA 16S rRNA genes were completely divided into five phylotypes, with the exception of one sequence derived from an adult patient with lung disease. This sequence differed from other adult-derived sequences by only one base. The five phylotypes were named IOLA PT1, PT2, PT3, PT4, and PT5. All sequences from adult lung disease patients, except for one sequence (CL22), belonged to PT1. The homology between PT1 and PT2 was 99.8%, and there were only three base differences out of 1,436 bases. PT4 and PT5 differed by 11 nucleotides (99.2%), but PT1, PT2, PT3, PT4, and PT5 each had 97% homology, indicating a large sequence difference (Fig. 3B). There were 88 IOLA-positive patients, of which 75 had only one IOLA-positive specimen and 13 had multiple IOLA-positive specimens. Of the 88 IOLA-positive patients, 53 had multiple visits; nasal discharge specimens were collected at each visit, and four patients were IOLA-positive in all specimens (Fig. 3C). IOLA was transiently detected in the remaining 49 patients. Of these, 18 patients changed from IOLA-PCR-positive to IOLA-PCR-negative (Fig. 3C). In the 2-year study, a single PT was detected consecutively in each IOLA-positive patient, and no patient had multiple PTs.

## DISCUSSION

In 2 years of surveillance in pediatric clinics across two Japanese cities with populations in excess of 900,000, the IOLA 16S rRNA gene was detected in 5.4% of nasal discharge specimens from children. The previously reported rate detected in BALF specimens derived from adult respiratory disease patients was 2.4% (12), indicating that IOLA is more frequently detected in nasal discharge from children than in BALF specimens from adult patients. Since there was no difference in the detection frequency of locations (cities) and no significant seasonal dependency in the IOLA-positive rate depending on the time of collection (season), IOLA is not considered to cause seasonal infectious diseases, such as influenza. We also revealed that sex and fever did not affect the detection of IOLA. Furthermore, the difference in IOLA detection frequency depending on the type of disease was unclear, suggesting that IOLA in nasal discharge may not directly affect the symptoms of these patients. Although pediatric nasal discharge reportedly contains representative pathogens of respiratory diseases (*Streptococcus pneumoniae*, *Haemophilus influenzae*, and *Moraxella catarrhalis*, etc) (16), these may not necessarily be the cause of the symptoms in children (17). Therefore, evaluating the relationship between bacteria detected in nasal discharge and symptoms proves challenging, as nasal discharge may not accurately represent the bacterial community at the site of infection. Furthermore, some studies have reported that pathogenic bacteria in nasal discharge might be involved in the horizontal transmission of respiratory diseases (18, 19). The age of IOLA-positive and IOLA-negative patients was significantly higher in positive patients. Although most specimens were collected from children aged ≤1 year (78.5%, 1,507/1,920), the positivity rate was significantly higher in children aged 2, 3, and 6 years. At 2–3 and 6 years of age, children start attending nursery and elementary schools, respectively, in Japan. These results suggest that IOLA is harbored in the nasal discharge of children and transmitted horizontally through contact with nasal secretions in a group.

The five phylotypes of IOLA discovered by phylogenetic analysis in this study were shown to be generally distributed in children’s nasal discharge without bias across cities. In this experiment, multiple IOLA-positive specimens were obtained from 13 out of the 88 patients, but no patients presented with multiple IOLA phylotypes. Nasal discharge samples from patients with IOLA were not always IOLA-positive, and IOLA detection was transient in most patients. Based on these results, we suggest that IOLA does not inhabit nasal discharge but transiently proliferates somewhere in nasal discharge or airways and then disappears. Furthermore, owing to the lack of clinical means to detect IOLA and its mild symptoms in patients, many IOLA-positive patients may be diagnosed with acute respiratory tract inflammation of unknown cause. Our findings reveal a significantly elevated detection rate of IOLA in patients with acute respiratory tract inflammation of unknown etiology than that in those with a definitive cause. Therefore, our findings suggest the potential involvement of IOLA in some respiratory tract inflammations of unknown etiology.

In our previous study (12), IOLA was continuously detected in the BALFs of an adult patient with chronic lower respiratory tract infections for at least 15 months. However, IOLA disappeared after treatment with an antibiotic (clarithromycin), and the patient did not subsequently develop chronic exacerbations of pneumonia. Eight of the 11 adult patients whose BALF was positive for IOLA had one or more comorbidities, and five of the eight patients were in an immunosuppressed state (12). IOLA likely multiplies in the respiratory tract of humans with immature immune systems, such as infants, and disappears transiently with asymptomatic or mild symptoms. However, in immunocompromised adults, IOLAs may colonize the respiratory tract and affect patients. The most notable fact is that one IOLA phylotype was found in 10 adult patients in the previous study, which was one (PT1) of the five IOLA phylotypes obtained in this study. One IOLA sequence derived from adult patients differed by one base (out of 1,435 bases) from the other 10 sequences, but this sequence was also the most similar to PT1. This strongly suggests that each IOLA lineage may have different effects on humans. The characteristics of IOLA strains even in the same phylotype may also differ, as a previous study showed that some of the genomic sequence regions in each of the 11 IOLA PT1 lineages obtained from adult patients are different (12). The interaction between IOLA and the host remains unknown, but it is clear that IOLA is not a common bacterium in the human body and is transmitted transiently during childhood.

This study has several limitations. First, since this study was conducted in only two Japanese cities, its representativeness is limited to Japanese children. In addition, the global distribution and genetic diversity of IOLAs remain unknown. IOLA has also been detected in patients in the USA, indicating that IOLA is not a bacterium endemic to Japan. Second, because we focused on patients with nasal discharge symptoms, the detection frequency of IOLA in healthy subjects without nasal discharge symptoms remains unknown. Collecting nasal discharge from healthy subjects is not feasible as they do not experience nasal discharge in a healthy state. Despite extensive research on the human microbiota, including the human respiratory tract, no registrations of IOLA-like sequences or reports regarding IOLA have been found. Furthermore, since the IOLA sequence has only been detected in patients with respiratory diseases and children with symptoms of nasal discharge, it is unlikely that IOLA is a resident bacterium in healthy humans. Third, as only 16S rRNA gene sequences were analyzed, the genomic diversity among the five IOLA phylotypes remains unknown. Owing to the extremely small genome size of IOLA, enrichment was essential to reconstruct its genome from a BALF sample containing large amounts of human cells and DNA. However, nasal discharge specimens, with higher viscosity and higher amounts of human cells and DNA than BALF samples, have been unsuccessful.

Due to its undetectability through Gram staining or standard culture methods and the unclear symptoms it presents, IOLA is rarely identified by clinicians. In the future, it is crucial to investigate the infectivity and pathogenicity of IOLA in humans by establishing a culture method and examining its properties. Elucidating the clinical significance of IOLA may be important for investigating respiratory diseases with unknown causes and may provide insights into the association between IOLA and chronic respiratory diseases.

## MATERIALS AND METHODS

### Participant enrollment

This study was approved by the Japanese University of Occupational and Environmental Health Medical Research Ethics Committee (No. H28-184). All experiments and methods in this study were conducted in accordance with relevant guidelines and regulations. The subjects were children who visited pediatric clinics in two cities (Clinic A: Kitakyushu City, with a population of approximately 0.96 million; and Clinic B: Fukuoka City, with a population of approximately 1.62 million) from January 2018 to December 2019 and who had symptoms of nasal discharge requiring aspiration. Nasal discharge specimens were sequentially collected biweekly at each pediatric clinic, with 20 specimens taken each time for a total of 40 specimens per month. When patients had multiple visits within a month, only the first sample was used. A specimen collection container was placed between the aspirator and the aspiration port, and the collected nasal discharge was used as the sample. All the specimens were stored at 4°C until further use. Clinical and demographic data were collected using a standardized questionnaire. Physicians or nurses collected clinical information including sex, age, body temperature, symptoms, and whether the patient had used antibiotics within 1 month of the visit. In addition, rapid antigen detection tests were performed for adenovirus (ImunoAce Adeno, TAUNS Laboratories Inc., Shizuoka, Japan), influenza virus (ImunoAce Flu, TAUNS Laboratories Inc.), human metapneumovirus (ImunoAce hMPV, TAUNS Laboratories Inc.), respiratory syncytial virus (ImunoAce RSV Neo, TAUNS Laboratories Inc.), and group A beta-hemolytic *Streptococcus* (Strep A test pack plus OBC kit, SANWA KAGAKU KENKYUSHO CO., LTD., Japan) as part of the clinically tested. This study was approved by the Japanese University of Occupational and Environmental Health Medical Research Ethics Committee (no. H28-184). All experiments and methods used in this study were conducted in accordance with relevant guidelines and regulations. As all subjects were children, written informed consent was acquired from their parents or legally authorized guardians.

### Diagnosis

The diagnoses were made by a pediatrician, and the final decision was made by two pediatricians based on medical records. Among the patients with respiratory symptoms such as fever, cough, expectoration, and dyspnea, those with new infiltrative findings on chest X-ray were diagnosed with acute pneumonia. When rapid antigen detection tests were positive, the patients were diagnosed with adenovirus, influenza, human metapneumovirus, respiratory syncytial virus, or group A streptococcal infections. Patients with respiratory symptoms that did not meet the above diagnostic criteria were diagnosed with acute respiratory tract inflammation.

### DNA extraction

As previously reported (15), 100 µL of nasal discharge specimen was mixed with 900 µL of phosphate-buffered saline, stirred, and homogenized using Micro Smash MS-100 (Tomy Seiko Co., Ltd. Tokyo, Japan). An aliquot (630 µL) of the suspension, 70 µL of 30% sodium dodecyl sulfate solution, and glass beads were mixed and then vigorously shaken. After treatment with TE-saturated phenol to this solution, DNA in the aqueous phase was washed and concentrated in 30 µL of TE buffer using an Amicon Ultra-100K filter (Merck Millipore Ltd., Billerica, MA, USA). DNA extracted and purified from 630 µL of nasal discharge was used for subsequent experiments. DNA extracted from phosphate-buffered saline (630 µL) only as above was used as a negative control for PCR.

### Detection of the IOLA 16S rRNA gene

To detect the IOLA 16S rRNA gene, IOLA-specific nested PCR was performed as previously described (12). This method can detect even a few copies of the gene and has been used to identify IOLA-positive samples in a large-scale survey of BALF specimens. The first PCR (10 µL in total volume) was performed using 1 µL of DNA solution, a universal bacterial primer set (E341f and E907r; each at a final concentration of 100 nmol/L), and AmpliTaq Gold DNA 360 Polymerase Master Mix (Thermo Fisher Scientific Inc., Waltham, MA, USA). The reaction condition was denaturing at 96°C for 5 min, followed by 25 cycles at 96°C for 30 s, 53°C for 30 s, and 72°C for 1 min, and a final extension step at 72°C for 2 min. After the first PCR, the reaction mixture was diluted 10-fold with TE buffer and was used as a template for the second PCR. The second PCR (10 µL in total volume) mixture was prepared as in the first PCR except for the primer set (IOLA-F1 and IOLA-R0). The reaction condition was achieved as described for the first PCR except that 30 reaction cycles were used. PCR products were confirmed using 2% agarose gel electrophoresis.

### Phylogenetic analysis of the IOLA 16S rRNA gene

The near full-length IOLA 16S rRNA gene was amplified using 1 µL of the DNA solution extracted from the IOLA-positive specimen as a template. The following primers were used: IOLA27F–5′-GAGTTTGATCCTGGCTCAG-3′ and IOLAnR1–5′-GTCAAAAGCGCAGGTTCAC-3′ (each at a final concentration of 100 nmol/L), and AmpliTaq Gold 360 were used to prepare a 20 µL reaction solution. The reaction mixture was incubated at 96°C for 5 min, 96°C for 30 s, 52°C for 30 s, 30 cycles of 2 min at 72°C, and a final incubation of 5 min at 72°C. Subsequently, 3 µL of the reaction mixture was electrophoresed using 1% agarose gel to confirm the amplified product of approximately 1,500 bp in length. Consequently, nested PCR was performed on samples with insufficient amplification. The second PCR was performed using 1 µL of the 10-fold diluted first PCR reaction solution with TE buffer as a template. The primers used were the aforementioned IOLA27F primer and IOLAnR2–5′-GTTCACCTACACTTACCTTG-3′ (each at a final concentration of 100 nmol/L). AmpliTaq Gold 360 was used to prepare a 20 µL reaction mixture, which was incubated at 5 min at 96°C, 30 cycles of 96°C for 30 s, 56°C for 30 s, and 72°C for 2 min. This was followed by a final incubation for 5 min at 72°C. Amplified products were confirmed using 1% agarose gel electrophoresis, as described above.

After primers and deoxyribonucleotide triphosphates (dNTPs) were eliminated using ExoSAP-IT (Thermo Fisher Scientific Inc.), sequencing reactions were performed using the BigDye Terminator Cycle Sequencing Kit v3.1 (Thermo Fisher Scientific Inc.) with 1 µL of each second PCR solution used as a template. The primers used were: E341F–5′-CCTACGGGAGGCAGCAG-3′, 926 f–5′-AAACTYAAAKGAATTGACGG-3′, IOLA530R–5′-CGCGGTTGCTGGCAC-3′, and IOLA1060R–5′-GAAGATGTAATTGCTTACAC-3′. The nucleotide sequences were determined in triplicate for each primer using a 3130xl Genetic Analyzer (Thermo Fisher Scientific Inc.). Assembly was performed using ATSQ5.1.3 (GENETYX Corp., Tokyo, Japan). For each assembly, we visually checked the waveform of each base and obtained sequences that contained no ambiguous bases (*N*). Phylogenetic analysis was performed based on the assembled sequences with a length of 1,436 bp. Multiple alignments were performed using MAFFT (20) with default settings. An ML tree was constructed with the HKY + F + I model selected using ModelFinder in IQ-TREE v2.2.0 (21). A phylogenetic tree was constructed using FigTree v1.4.4 (http://tree.bio.ed.ac.uk/software/figtree/). All IOLA-16S rRNA gene sequences (accession number: LC759651–LC759753) determined in this study were deposited in the DNA Data Bank of Japan (DDBJ)/European Molecular Biology Laboratory (EMBL)/GenBank.

### Statistical analysis

Bellcurve for Excel v4.02 (https://bellcurve.jp/ex/) was used to compare categorical variables via the Mann–Whitney U test, χ^2^ test, and residual analysis, as appropriate. In this study, a two-sided *P* value less than 0.05 was considered statistically significant.

## Data Availability

The 16S rRNA gene sequences of IOLA from the BALFs of adult patients (accession no. LC435447–LC435456) and from the children's nasal discharges (accession no. LC759651–LC759753) have been deposited in DDBJ/EMBL/GenBank.

## SUPPLEMENTAL MATERIAL

Supplemental material spectrum.00566-24-s0001.xlsx Table S1.

ASM does not own the copyrights to Supplemental Material that may be linked to, or accessed through, an article. The authors have granted ASM a non-exclusive, world-wide license to publish the Supplemental Material files. Please contact the corresponding author directly for reuse.
